# Conserved Inhibitory Mechanism and Competent ATP Binding Mode for Adenylyltransferases with Fic Fold

**DOI:** 10.1371/journal.pone.0064901

**Published:** 2013-05-30

**Authors:** Arnaud Goepfert, Frédéric V. Stanger, Christoph Dehio, Tilman Schirmer

**Affiliations:** 1 Focal Area Infection Biology, Biozentrum, University of Basel, Basel, Switzerland; 2 Focal Area Structural Biology and Biophysics, Biozentrum, University of Basel, Basel, Switzerland; Centre National de la Recherche Scientifique, Aix-Marseille Université, France

## Abstract

The ubiquitous FIC domain is evolutionarily conserved from bacteria to human and has been shown to catalyze AMP transfer onto protein side-chain hydroxyl groups. Recently, it was predicted that most catalytically competent Fic proteins are inhibited by the presence of an inhibitory helix α_inh_ that is provided by a cognate anti-toxin (class I), or is part of the N- or C-terminal part of the Fic protein itself (classes II and III). *In vitro*, inhibition is relieved by mutation of a conserved glutamate of α_inh_ to glycine. For the class III bacterial Fic protein NmFic from *Neisseria meningitidis*, the inhibitory mechanism has been elucidated. Here, we extend above study by including bacterial class I and II Fic proteins VbhT from *Bartonella schoenbuchensis* and SoFic from *Shewanella oneidensis*, respectively, and the respective E->G mutants. Comparative enzymatic and crystallographic analyses show that, in all three classes, the ATP substrate binds to the wild-type FIC domains, but with the α-phosphate in disparate and non-competent orientations. In the E->G mutants, however, the tri-phosphate moiety is found reorganized to the same tightly bound structure through a unique set of hydrogen bonds with Fic signature motif residues. The γ-phosphate adopts the location that is taken by the inhibitory glutamate in wild-type resulting in an α-phosphate orientation that can be attacked in-line by a target side-chain hydroxyl group. The latter is properly registered to the Fic active center by main-chain β-interactions with the β-hairpin flap. These data indicate that the active site motif and the exposed edge of the flap are both required to form an adenylylation-competent Fic protein.

## Introduction

Adenylyl transferases (ATases) utilize adenosine triphosphate (ATP) to covalently modify proteins, nucleic acids, or small molecules with adenosine monophosphate (AMP), a reaction known as adenylylation or AMPylation. The ubiquitous FIC domain (pfam 02661) found in proteins of all domains of life and viruses has only recently been shown to confer ATase activity. Thus, the bacterial T3SS effector protein VopS from *Vibrio parahaemolyticus* and the surface antigen IbpA from *Histophilus somni* covalently attach the bulky AMP moiety onto a specific threonine or tyrosine, respectively, of the switch I region of Rho family GTPases [1], [2]. This abrogates binding of downstream effectors and results in actin cytoskeleton collapse and concomitant cell detachment and death. Mutational and bioinformatics analysis indicated that Fic proteins containing a strictly conserved HxFx(D/E)GNGRxxR signature motif in the active center typically display adenylylation activity [1], [2], [3], [4], [5], while Fic proteins with an active center deviating from this consensus are considered to have adopted different activities. Indeed, the host-targeted effector protein AnkX of *Legionella pneumophila* exhibiting an HxFxDANGRxxV signature motif displays phosphocholination activity towards the GTPase Rab1 [6].

The FIC domain is structurally characterized by a conserved central core of four helices (α2 to α5) that is flanked by three helices (α1, α6 and α7) found in diverse dispositions in different Fic proteins [3], [7]. Helices α4 and α5 are joined by a loop that together with the N-terminal cap of helix α5 forms the active center represented by a signature motif with the consensus sequence HxFx(D/E)GNGRxxR. The catalytic mechanism of adenylylation was deduced from the crystal structure of the second FIC domain of IbpA in complex with the adenylylated Cdc42 target [4] and from biochemical studies [5] and shown to involve nucleophilic attack of the target side-chain hydroxyl onto the ATP α-phosphate. The triphosphate binding site at the anionic nest at the N-terminus of helix α5 was characterized by the crystal structure of BepA from *Bartonella henselae* in complex with pyrophosphate, the side product of the reaction [3]. An ATP substrate complex structure was obtained recently for the Fic protein of *Neisseria meningitidis* [8] corroborating the catalytic mechanism. The histidine of the signature motif is critical for deprotonation of the incoming target hydroxyl group [5], whereas the phenylylanine is part of the hydrophobic core of the domain. The remaining residues of the motif are involved in ATP/Mg^2+^ binding and loop stabilization [3], [8].

We recently demonstrated that the Fic protein VbhT from *Bartonella schoenbuchensis* causes bacterial growth arrest when overexpressed in *Bartonella* or *E. coli* and that this effect can be repressed by co-expression with the anti-toxin VbhA, a small protein encoded upstream of VbhT [8]. As shown by structure analysis, VbhA forms a tight complex with the FIC domain of VbhT with the conserved glutamate (E_inh_) from the inhibitory helix α_inh_ partly obstructing the ATP binding site, which gave a first clue regarding the inhibitory mechanism mediated by VbhA binding.

Exhaustive bioinformatic analysis coupled with homology modeling revealed that the (S/T)xxxE(G/N) signature motif of α_inh_ is not only found in several other putative anti-toxin sequences coded immediately upstream of Fic proteins, but is often part of the FIC domain itself either preceding helix α1 or immediately following helix α7 [8]. Thus, a classification system was introduced grouping the Fic proteins for which an anti-toxin with an inhibitory helix α_inh_ had been found into class I and those with an equivalent of α_inh_ in the N- or C-terminal part of the Fic protein into classes II and III, respectively. Indeed, 90% of the Fic proteins with the canonical FIC signature motif could be classified accordingly, suggesting that all these enzymes are inhibited in their enzymatic activity.

The physiological stimulus or condition for relief of α_inh_-mediated inhibition is not yet known. For T4SS Fic proteins of class I (such as VbhT or BepA [9]), however, it appears likely that, for injection into host cells, the Fic protein has to unfold and will be translocated without the antitoxin. For class II and III proteins, detachment, unfolding, or proteolytic cleavage of the α_inh_ helix may cause relief of inhibition. In fact, a truncation mutant of the class III Fic protein from *N. meningitidis* (NmFic) lacking the entire C-terminal α_inh_ helix showed strong ATase activity and allowed to study the catalytic and inhibitory mechanism in detail [8]. A more subtle means to relieve inhibition, which is applicable to Fic proteins of all three classes, is the replacement of the inhibitory glutamate by glycine. *In vivo*, such E->G mutations showed a detrimental effect on bacterial growth [8]. For the human HYPE protein (class II), the corresponding mutant protein catalyzed *in vitro* AMP transfer to the small GTPases Rac1 and Cdc42, whereas only marginal effect was seen with the wild-type proteins [8].

Here, we assayed in a systematic approach Fic representatives of the three Fic classes and their E->G mutants for *in vitro* adenylylation showing that the mutation causes inhibition relief across the Fic classes. Binding of ATP substrate or AMPPNP substrate analog to the wild-type and the E->G mutant proteins was studied by protein crystallography to reveal the inhibitory mechanism and to get further insight into catalysis. This yielded a consistent molecular mechanism that most likely applies to most adenylylation competent Fic proteins irrespective of class.

## Materials and Methods

### Cloning

The full-length *vbhA* gene and part of the *vbhT* gene (amino acid residues 1–248, His_6_-tagged) were amplified from plasmid pPE0021 and cloned into the pRSF-Duet1 vector leading to plasmid pAG0077 (VbhA/VbhT(FIC)). The full-length *vbhA* gene and part of the *vbhT* gene encoding the FIC domain (amino acid residues 1–198, His_6_-tagged) were PCR-amplified from plasmid pPE0021 and cloned into the pRSF-Duet1 vector (pFVS0011). A two-base pair mutation is then introduced in pFVS0011 to obtain plasmid pFVS0065 (VbhA_E24G_/VbhT(FIC)). The *fic* gene of *Neisseria meningitidis* was PCR-amplified with an N-terminal His_6-_tag from *Neisseria meningitidis* from coding region of amino acid residues 11–191 to generate plasmid expressing NmFic (pFVS0015). The E186G mutant construct (NmFic_E186G_, pFVS0059) was generated by introducing a two-base pair mutation in pFVS0015. The *fic* gene of *Shewanella oneidensis* was PCR-amplified from plasmid (ASU biodesign institute, Clone ID SoCD00104192) and cloned with an N-terminal His_6_-tag into pRSF-Duet1 (pFVS0040). The SoFic_E73G_ plasmid (pFVS0058) was generated by introducing a two-base pair point mutation in pFVS0040.

### Protein Expression and Purification

Vectors pAG0077 (VbhA/VbhT(FIC)), pFVS0040 (SoFic) and pFVS0015 (NmFic) were transformed into *E.coli* BL21 (DE3). *E. coli* cultures were grown at 37°C in LB medium supplemented with 50 µg/ml of kanamycin to an OD_595_ of 0.6 before induction with 0.3 mM IPTG for 16 h at 23°C. Vectors pFVS0065 (VbhA_E24G_/VbhT(FIC)), pFVS0059 (NmFic_E186G_), pFVS0058 (SoFic_E73G_) were transformed into BL21-AI cells**.** Cells were incubated in 750 ml LB medium supplemented with 50 µg/ml kanamycin and 1% glucose at 37°C at 200 rpm until an OD_595_ value of 1.5 was reached. Bacterial pellets were resuspended in 1 L of Terrific Broth media containing 50 µg/ml^−1^ kanamycin. Protein expression was induced at 23°C with 0.1% arabinose and 0.1 mM IPTG for 23 h at 200 rpm.

Cells containing overexpressed VbhA/VbhT(FIC) and NmFic were resuspended in lysis buffer containing 20 mM Tris (pH 7.5), 250 mM NaCl, and 25 mM imidazole and disrupted using French press. Cell debris were pelleted by ultracentrifugation and the supernatant was applied to a His-Trap column (GE Healthcare). The proteins were eluted with a gradient of elution buffer containing 20 mM Tris (pH 7.5), 250 mM NaCl, and 500 mM imidazole. The proteins were then concentrated and injected on a Superdex 75 16/60 gel filtration column (GE Healthcare) equilibrated with 10 mM Tris (pH 7.6) and 100 mM NaCl. The pure proteins were concentrated to 3.7 mg/ml for VbhA/VbhT(FIC) and 30 mg/ml for NmFic.

The same purification protocol as described above was used for VbhA_E24G_/VbhT(FIC) and NmFic_E186G_ with an additional intermediate purification step. After affinity purification, the proteins were adjusted to 20 mM Tris (pH 8.5), 25 mM NaCl, applied to a Resource-Q anion exchange column (Amersham Biosciences), and eluted with a linear gradient of 1 M NaCl. Peak fractions were concentrated and further purified by gel filtration chromatography. Purified proteins in 10 mM Tris (pH 7.6), 100 mM NaCl were concentrated to 4.1 mg/ml for VbhA_E24G_/VbhT(FIC) and 33 mg/ml for NmFic_E186G_. Cells containing overexpressed SoFic and SoFic_E73G_ were resuspended in lysis buffer containing 50 mM HEPES (pH 8.0), 50 mM NaCl, 1 mM TCEP, 10% glycerol and 10 mM Imidazole and disrupted using French press. Cell debris were pelleted by ultracentrifugation and the supernatant was applied to a His-Trap column (GE Healthcare). The proteins were eluted with a gradient of elution buffer containing 50 mM HEPES (pH 8.0), 50 mM NaCl, 1 mM TCEP, 10% glycerol and 300 mM imidazole. The proteins were then concentrated and injected on a Superdex 75 16/60 gel filtration column (GE Healthcare) equilibrated with 20 mM HEPES (pH 8.0), 200 mM NaCl and 1 mM TCEP. The pure proteins were concentrated to 21.8 mg/ml for SoFic and 12 mg/ml for SoFic_E73G._

### Protein Crystallization

For crystallization, the hanging-drop vapor diffusion method was used with 1 µl protein solution mixed with 1 µl reservoir solution. The VbhA/VbhT(FIC) and VbhA_E24G_/VbhT(FIC) complexes were concentrated to 3.7 mg/ml and 4.1 mg/ml, respectively, and crystallized at 20°C using a reservoir solution composed of 15% (w/v) PEG 4000, 0.1 M MES pH 6.5. Whereas, the wild-type crystal was soaked with 5 mM ATP, and 5 mM MgCl_2_, the mutant was co-crystallized with 10 mM ATP, and 10 mM MgCl_2_. For data collection, crystals were transferred to reservoir solutions supplemented with 20% glycerol and flash frozen in liquid nitrogen. SoFic and SoFic_E73G_ were concentrated to 21.8 mg/ml and 12 mg/ml, respectively, and co-crystallized with either 5 mM ATP or 5 mM AMPPNP and supplemented with 5 mM MgCl_2_ in a solution composed of 21% (w/v) PEG 3350 and 0.2 M NaF pH 7.1 at 4°C. For data collection, crystals of the protein-ligand complex were cryoprotected by transfer to a reservoir solution supplemented with 15% (v/v) PEG 200 and flash cooled in liquid nitrogen. For crystallization of NmFic_E186G_ (33 mg/ml), a reservoir solution composed of 4 M potassium formate, 0.1 M Bis-Tris propane pH 9.0, 2% (w/v) PEG MME 2000 was used. Crystals were soaked with 5 mM AMPPNP and 5 mM MgCl_2_ and then cryoprotected with 20% glycerol prior flash-cooling in liquid nitrogen.

### Data Collection, Structure Determination, and Refinement

Diffraction data were collected at the Swiss Light Source at 100 K and processed using XDS [10]. The structures were solved by molecular replacement using the apo structures of VbhA/VbhT(FIC) (PDB code 3SHG), SoFic (PDB code 3EQX) or NmFic (PDB code 2G03) as search models using Phaser [11]. Several rounds of iterative model building and refinement were performed using Coot [12] and PHENIX [13] or REFMAC5 [14], respectively. 5% of the data were excluded from refinement and used for cross-validation. The geometry of the final model was assessed using MolProbity [15] showing >99% of the residues in the core and allowed regions of the Ramachandran plot. Data collections and refinement statistics are summarized in Table 1. The atomic coordinates and structure factors of VbhA/VbhT(FIC)/ATP, VbhA_E24G_/VbhT(FIC)/ATP, SoFic/ATP, SoFic_E73G_/AMPPNP, and NmFic_E186G_/AMPPNP have been deposited in the Protein Data Bank under accession codes 3ZC7, 3ZCB, 3ZCN, 3ZEC and 3ZLM, respectively. The figures were generated with Dino (A. Philippsen unpublished, http://www.dino3d.org).

**Table 1:** Data collection and refinement statistics.

Protein	VbhA/VbhT(FIC)	VbhA_E24G_/VbhT(FIC)	SoFic	SoFic_E73G_	NmFic_E186G_
Ligand	ATP	ATP	ATP	AMPPNP	AMPPNP
PDB code	3ZC7	3ZCB	3ZCN	3ZEC	3ZLM
**Data collection**					
Wavelength (Å)	1.000	0.979	0.979	0.979	1.000
Detector	MAR225 CCD	PILATUS 2M	MAR225 CCD	MAR225 CCD	PILATUS 2M
Space group	C2	C2	P2_1_	P2_1_2_1_2_1_	P6_4_22
Cell dimensions					
*a*, *b*, *c* (Å)	106.5, 40.6, 73.7	106.5, 40.3, 73.9	37.8, 164.9, 70.2	71.3, 80.6, 141.8	149.1, 149.1, 76.4
*α β γ* (°)	90.0, 121.6, 90.0	90.0, 121.4, 90.0	90.0, 94.4, 90.0	90.0, 90.0, 90.0	90.0, 90.0, 120.0
Resolution (Å)	45.4−2.1 (2.2−2.1)	45.4−1.9 (2.1−1.9)	35.5−1.7 (1.8−1.7)	42.7−2.2 (2.3−2.2)	49.3−2.0 (2.1−2.0)
*R*_sym_ or *R*_merge_ (%)	8.5 (33.8)	5.7 (33.4)	4.4 (41.3)	10.5 (52.8)	6.3 (72.7)
CC(1/2) (%)	99.8 (93.6)	99.9 (93.1)	99.9 (87.1)	99.8 (90.3)	100.0 (97.9)
*I*/*σ*	18.9 (5.7)	14.5 (3.4)	22.9 (3.4)	17.6 (4.5)	31.1 (5.7)
Completeness (%)	99.2 (92.3)	99.2 (97.0)	99.5 (96.7)	100.0 (100.0)	99.9 (100.0)
Multiplicity	5.4 (4.9)	3.6 (3.6)	3.9 (3.4)	7.4 (7.5)	21.4 (22.6)
**Refinement**					
Resolution (Å)	15.0−2.10	30.0−1.94	15.0−1.70	15.0−2.20	30.0−2.00
No. reflections	15,769 (2,342)	18,923 (1,355)	93,100 (2,837)	42,085 (3,956)	32,490 (2,338)
*R*_work/_ *R*_free_ [%]	16.6/23.0	19.4/23.4	16.7/20.2	16.5/21.1	18.2/19.9
Mol./a.u	1	1	2	2	1
No. atoms					
Protein	2172	2011	5961	5984	1458
Ligand/ion	1 ATP	1 ATP, 1 MG	2 ATP	2 ANP, 1 MG	1 ANP, 1 MG
Water	226	134	981	695	135
Average B (Å^2^)					
Protein	22.0	25.0	21.9	20.7	45.9
Ligand/ion	39.7	27.5/11.4	21.9	12.9/28.9	40.1/64.8
Water	28.4	32.1	33.7	28.0	49.3
R.m.s deviations					
Bond lengths (Å)	0.007	0.011	0.008	0.009	0.010
Bond angles (°)	1.0	1.3	1.2	1.2	1.2

Values for the highest resolution shell are shown in brackets.

### *In vitro* Adenylylation Assay

Adenylylation activity of VbhA/VbhT(FIC), SoFic and NmFic constructs was assessed by incubating 125 ng, 1.25 µg and 2.5 µg of purified protein, respectively, with 10 µCi α-^32^P-ATP (Hartmann Analytic) in a buffer containing 50 mM Tris pH 8.0, 150 mM NaCl, 0.1 mM EGTA, 15 mM MgCl_2_, and protease inhibitor cocktail (Roche). Reactions were incubated for 1 h at 30°C, resolved by SDS–PAGE, and subjected to autoradiography.

## Results

### Constitutive Inhibition is Relieved by Truncation of the Inhibitory Glutamate in All Three Fic Classes

For the comparative structure/function study on the inhibitory mechanism of Fic proteins from the various classes we chose as representatives the FIC domain of VbhT (residues 1 to 198) from *Bartonella schoenbuchensis* in complex with its cognate antitoxin VbhA (VbhA/VbhT(FIC); class I), Fic protein SO_4266 *from Shewanella oneidensis* (SoFic; class II) and Fic protein NMB0255 from *Neisseria meningitidis* (NmFic; class III).

**Figure 1:**
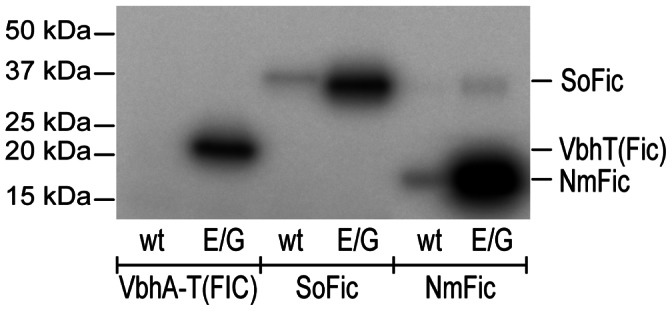
AMP transfer catalyzed by Fic proteins and their inhibition-relieved variants. Autoradiography of VbhA/VbhT(FIC), SoFic and NmFic (wt, wild type; E/G, E->G mutant) after incubation with radioactively labeled α-^32^P-ATP.

Auto-adenylylation is a convenient read-out to assess adenylylation activity of Fic proteins. It does not require the presence of a physiological protein target that may, in fact, not yet been known as in the case of SoFic. Autoradiographies of SDS-PAGE gels after incubation with α-^32^P-ATP (Fig. 1) show that auto-adenylylation is virtually absent in the wild-type Fic proteins of all three classes, i.e. for VbhA/VbhT(FIC), SoFic, and NmFic (see also ref. 8), but is drastically boosted in the respective E->G mutants suggesting a common inhibitory mechanism.

### ATP Binds to Wild-type Fic Proteins in Disparate and Catalytically Incompetent Conformations

**Figure 2:**
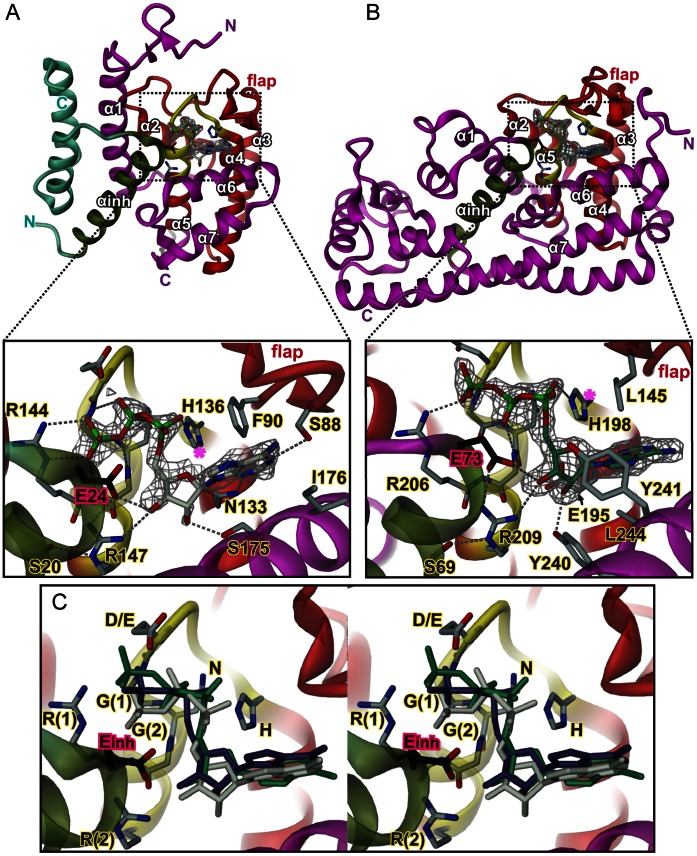
Crystal structures of wild-type Fic proteins representing classes I to II in complex with ATP substrate. (A) VbhA/VbhT(FIC), (B) SoFic. Structures are shown in cartoon representation (red, FIC core as defined by PFAM; yellow, active site loop and N-terminal end of helix α5; dark-green, inhibitory helix α_inh_). In (A), the fold of the antitoxin is shown in dark-green and steel-blue. Selected residues are shown in full with the inhibitory glutamate (E24 or E73, respectively) colored in dark. The 2Fo-Fc simulated annealing omit maps covering the ligand are contoured at 1.1 σ. In both structures, the orientation of the α-phosphate prevents nucleophilic attack of a putative target side-chain hydroxyl onto the ATP substrate, since the position inline with the scissile Pα-O3α bond (magenta star) is unattainable. C) Stereo view of the superposition of the ATP nucleotides shown in panel A and B with AMPPNP from the complex structure of the class III NmFic protein (PDB 3S6A [8]) within the active site of the VbhA/VbhT(FIC) complex (same as in panel A). The nucleotides of the various complexes are distinguished by their colors (white for the ATP bound to VbhA/VbhT(FIC), green for the ATP bound to SoFic, and blue for the AMPPNP of the NmFic complex. Note that the AMPPNP γ-phosphate in NmFic is found disordered [8] and therefore not shown. The residues of the HxFx(D/E)GNGRxxR Fic signature motif are labeled, the two glycine and the two arginine residues are distinguished by a "1" or "2" in brackets. The phenylalanine (not shown) is part of the hydrophobic core. The inhibitory glutamate from α_inh_ is labeled as E_inh_.

Fig. 2 shows the high-resolution structures of VbhA/VbhT(FIC) (class I) and SoFic (class II), both in complex with ATP. Whereas VbhA/VbhT(FIC) crystallized isomorphously to the unliganded wild-type crystals ([8], PDB code 3SHG), SoFic yielded crystals of monoclinic space group, i.e. distinct to the orthorhombic form of the apo structure ([16], PDB code 3EQX). In the two structures the nucleotide is clearly visible, albeit with elevated B-factors (40 Å^2^) in VbhA/VbhT(FIC). Only marginal structural changes are induced upon substrate binding (rms deviations between the Cα-positions of apo and complex form of 0.4 Å and 0.8 Å for VbhA/VbhT(FIC) and SoFic, respectively).

In both structures the ATP substrate is found at analogous sites (Fig. 2) with the base filling a pocket formed by α4, α6, and the β-hairpin flap, the ribose 3′-hydroxyl H-bonded to the conserved glutamate of α_inh_, and the triphosphate moiety interacting with the anionic nest formed by the N-terminus of α5. The same binding mode has been observed for class III NmFic [8]. In all three structures, also the ribose 2′-hydroxyl is forming an H-bond, but to non-homologous protein side-chains. Similarly, the binding sub-site for the base is not conserved on the residue level. However, in each case, hydrophobic residues are contributed by helix α6 and by the flap. A weak H-bond is formed between the adenine N3 and N133 in VbhT(FIC). A homologous interaction (with N104) is found in NmFic [8].

Most relevant for catalysis is the orientation of the α-phosphate that has to be accessible for nucleophilic attack by the target side-chain hydroxyl group. In VbhA/VbhT(FIC) and SoFic, as in NmFic [8], the position that is in-line with the scissile Pα-O3α bond is not accessible for an attacking group (Fig. 2). Such a group positioned there would severely clash with atoms of the enzyme. Thus, in Fic proteins of all three classes, catalytically non-competent orientation of the α-phosphate appears to be the reason for the lack of adenylylation activity.

Interestingly, while the α-phosphate is locked in a secured position in each of the structures, it shows distinct orientations among the three proteins that can be traced back to differences in the binding mode of the β- and γ-phosphates (Fig. 2C). Though interacting with the same protein groups (anionic nest; histidine, asparagine, and first arginine of the signature motif), the detailed H-bonding patterns are different (e.g. the main chain amide of the second glycine of the motif interacts with the bridging O3β in VbhA/VbhT(FIC), and with the non-bridging O1β in SoFic).

It seems that during convergent evolution of α_inh_-mediated adenylylation inhibition in the different Fic protein classes no strict constraints for the ATP binding mode were operational apart from the requirement for a non-competent orientation for the reacting phosphate.

### Truncation of the Inhibitory Glutamate Allows the ATP Substrate to Bind in a Catalysis Competent Conformation

Relief of Fic protein inhibition was achieved previously by expression of VbhT without its cognate antitoxin VbhA or by replacing in NmFic the SxxxE inhibition motif by AxxxA or – most drastically - by deleting the entire α_inh_ [8]. The conserved glutamate of α_inh_, E_inh_, was identified to be crucial for the inhibitory effect, since mere truncation of its side-chain (E->G mutation) rendered recombinantly overexpressed Fic proteins of all three classes toxic to *E. coli* [8].

*In vitro,* the mutation has a drastic effect in that auto-adenylylation is boosted in all three representative Fic proteins (Fig. 1). This opens the door for studying the action of any active Fic protein *in vivo*, even without knowing the physiological stimulus for inhibition relief.

**Figure 3:**
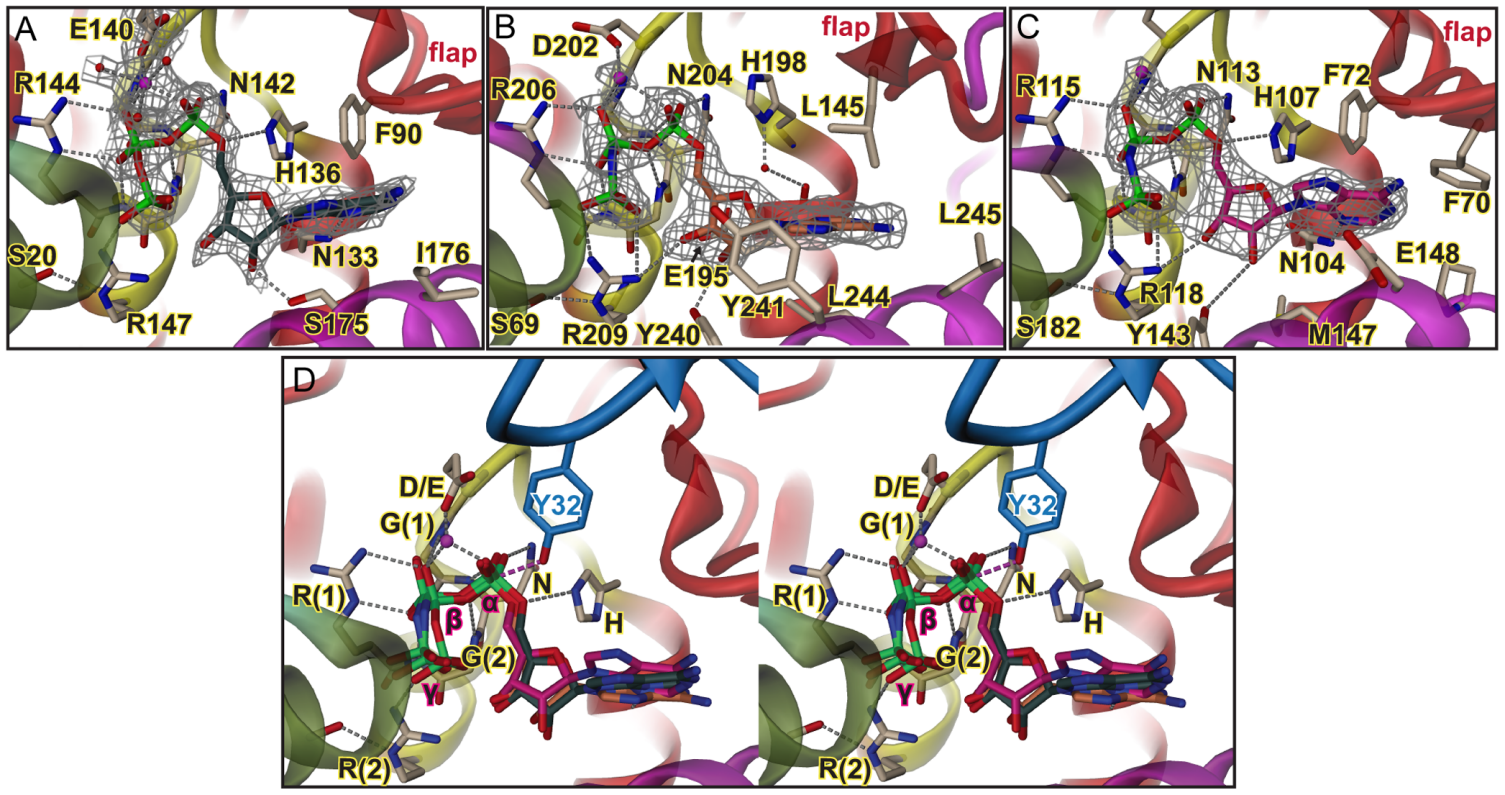
Crystal structures of E->G mutated Fic proteins representing classes I to III in complex with substrate or substrate analog. A, VbhA_E24G_/VbhT(FIC) in complex with ATP/Mg^2+^; B, SoFic_E73G_, C, NmFic_E186G_, both in complex with AMPPNP/Mg^2+^. Representation as in Fig. 2 with magnesium ions shown as magenta spheres. The 2Fo-Fc simulated annealing omit maps covering the nucleotide/Mg^2+^ ligands are contoured at 1.1 σ. D, Stereo view of the superposition of the ligand structures shown in panels B and C onto the VbhA_E24G_/VbhT(FIC) complex (same as in panel A). Note that the nucleotides of the various complexes are distinguished by their carbon color (VbhA_E24G_/VbhT(FIC) ATP in green, SoFic_E73G_ AMPPNP in orange and NmFic_E186G_ AMPPNP in pink). The residues of the HxFx(D/E)GNGRxxR signature motif are labeled as in Fig. 2C with the phenylalanine not shown. Also shown is the modifiable hydroxyl side-chain Y32 of Cdc42 (blue) after superposition of the IbpA(FIC2)/Cdc42 complex [4] onto VbhA_E24G_/VbhT(FIC). For the superposition, only the Fic active site loops were used. The α-phosphate moieties appear well-suited for in-line attack of the target hydroxyl group (broken line in magenta).

To reveal the underlying inhibition relief mechanism, crystal structures of the three mutant proteins in complex with ATP or AMPPNP were determined to high resolution. Although, in solution the mutants show auto-adenylylation, no such modification is observed in the crystal structures. For NmFic_E186G_ this is not surprising, since the complex structure has been obtained by soaking and auto-adenylylation would require partial unfolding of α_inh_ carrying the modifiable tyrosine (Y183) [8]. The VbhA_E24G_/VbhT(FIC) and SoFic_E73G_ complexes were co-crystallized. Since we do not see adenylylated residues, the extend of modifications may be either minor, locate to flexible loops or only the unmodified fraction may have crystallized. Figs. 3A–C show that in all three cases, the nucleotide is well resolved and, in contrast to the wild-type complexes, shows a unique conformation and relative position within the binding site (Fig. 3D).

While the base and ribose moieties interact with the mutant in the same way as with the wild-type proteins (compare with Fig. 2, see also Fig. 3a in [8] for NmFic), the triphosphate has adopted a strongly curved conformation with the terminal γ-phosphate approaching closely the ribose moiety and forming a tight salt-bridge with the second arginine of the FIC motif (R(2): R147, R209, or R118, respectively).

The position and orientation of the triphosphate is defined by a multitude of specific interactions (Fig. 3A–C). In all structures, the α- and β-phosphate moieties form four H-bonds with the four exposed backbone amide groups of the compound anion binding nest [17] at the N-terminal end of helix α5. In addition, the first arginine of the signature motif (R(1): R144, R206, or R115, respectively) forms a salt-bridge with the β-phosphate involving two H-bonds and the asparagine of the motif (N142, N204, or N113, respectively) interacts with a non-bridging oxygen of the α-phosphate.

In all the structures, a magnesium ion is present albeit with high B-factor for NmFic_E186G_ (63 Å^2^). The metal bridges the α- and β-phosphate and is coordinated in addition by the conserved D/E residue of the Fic signature motif in VbhA_E24G_/VbhT(FIC) and SoFic_E73G_ (E140, D202, respectively). It is particularly well resolved in the former structure where three well-defined water molecules complete its octahedral coordination shell (Fig. 3A). Interestingly, the divalent cation is observed only in the adenylylation competent complexes, but not in the wild-type complexes. Indeed, magnesium is indispensable for Fic mediated ATase activity (data not shown) and is probably important for fine-tuning of the α- and β-phosphate orientation within the compound anion binding nest and for stabilization of the transition state.

**Figure 4:**
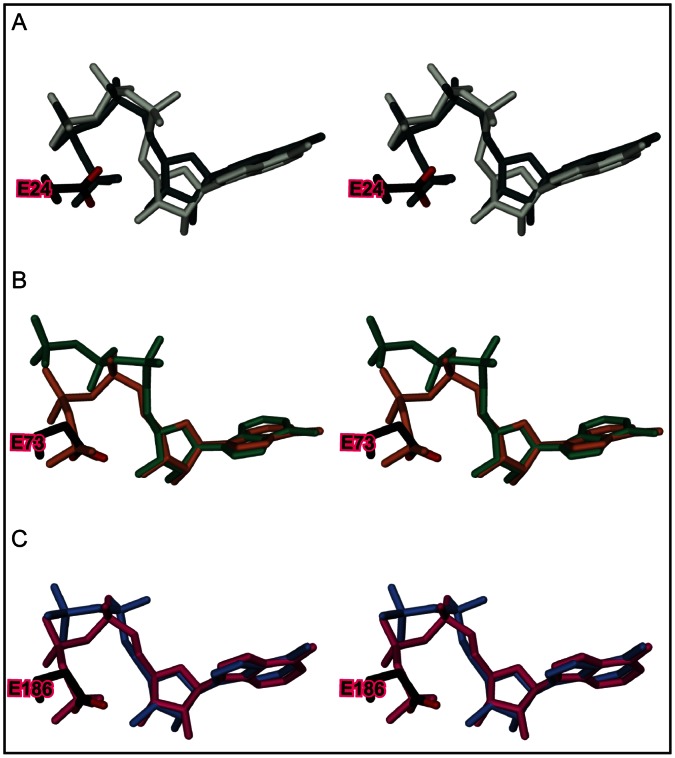
Comparison of triphosphate nucleotide structures as bound to wild-type and E->G mutated Fic proteins from class I to III. Stereo views of the ligand structures after superposition of the FIC domains (not shown). Also shown is the inhibitory glutamate of the wild-type structures. A, ATP as bound to VbhA/VbhT wild-type (white) and the E24G mutant (dark green). B, ATP and AMPPNP as bound to SoFic wild-type (green) and the E73G mutant (orange), respectively. C, AMPPNP as bound to NmFic (blue) and the E186G mutant (pink). Note that the AMPPNP γ-phosphate in NmFic is found disordered [8] and therefore not shown.

Overall, the three structures display a unique mode of ATP binding that can be attained only in the mutants, since the γ-phosphate effectively adopts the position that is taken by the inhibitory glutamate in the wild-type proteins (Fig. 4). Most relevantly, the reorganization of the triphosphate in the binding site results in a α-phosphate orientation that is now prone for in-line attack by an incoming target side-chain (Fig. 3D). Clearly, the conservation of this binding mode across the FIC classes shows that it is essential for FIC function.

### Target Registration to the FIC Active Site

**Figure 5:**
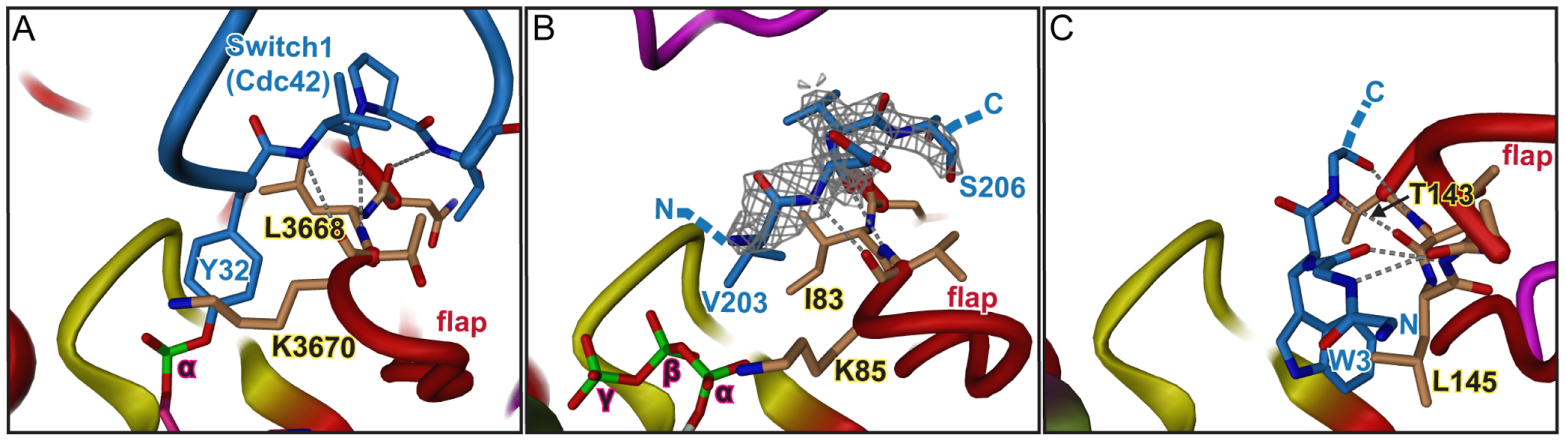
Sequence independent registration of peptide or target protein to the FIC flap. The bound peptide/protein segment (blue) and the target dock (brown) are shown in full. Main chain-main chain H-bonds are depicted as stippled lines. A, Product complex of IbpA(Fic2) with Cdc42 target [4]. Tyrosine 32 from the switch1 region of Cdc42 is adenylylated. B, VbhA/VbhT(FIC) complexed with residues 203 to 206 of a symmetry related molecule. The 2Fo-Fc simulated annealing omit map covering the residues 203 to 206 is contoured at 1.1 σ. Note that the preceding 7 residues are disordered and not shown. C, SoFic complexed with residues 0 to 4 of a symmetry related molecule (PDB 3EQX) [16]. The side-chains of residues 0, 1 and 4 are disordered and not displayed for clarity reason. Note that Y32 in panel A, V203 in panel B and W3 in panel C are in equivalent positions.

The conservation of the FIC active site and the ATP substrate binding mode prompts for a precise alignment of the incoming side-chain hydroxyl with the scissile Pα-O3α bond. The beta-hairpin flap partly covering the active site appears to represent a "target dock" that ensures this precise positioning of the target backbone stretch immediately following the modifiable hydroxyl side-chain and thus registers the side-chain to the active site as has been proposed before (2). This was deduced mainly from the only known Fic protein/target complex structure IbpA(FIC)/Cdc42 [4] where the AMPylated Y32 of Cdc42 is part of a segment (switch 1 loop) in extended conformation and complements inter-molecularly the β-hairpin of the flap (Fig. 5A).

This notion is further corroborated by the structure of the wild-type VbhA/VbhT(FIC) complex presented here that revealed additional density close to the flap above the active site (Fig. 5B). This was interpreted as a four residue peptide in extended conformation that is associated antiparallely to the edge of the two-stranded β-hairpin of the flap via three main chain-main chain H-bonds. Location and side-chain densities are consistent with the peptide representing residues 203 to 206 of a symmetry mate (note that the ordered part of the VbhT(FIC) construct ends with residue F197). Very similarly, peptide density is present at an equivalent location in the A-chain of SoFic_E73G_ and could be attributed to the N-terminus (residues 0 to 3) of a symmetry related B-chain as also reported for the isomorphous crystal structure of wild-type SoFic (Fig. 5C) [16].

Comparison of Figures 5A–C suggests that a tyrosine instead of the valine in position 203 of the symmetry related VbhT(FIC) chain or of the tryptophan in position 3 of the symmetry related SoFic chain would indeed be well poised to attack the ATP α-phosphate. Furthermore, it has been shown for IbpA that the side-chains of the target dock residues Leu3668 and Lys3670 form a hydrophobic clamp that fix the target tyrosine side-chain (Fig. 5C) [4]. Side-chains of residues I83 and K85 in VbhT(FIC) and residues T143 and L145 in SoFic, that hold the valine and tryptophan, respectively, may in a similar way clamp down the modifiable side-chain (Fig. 5A–B). Taken together, these observations show that the flap has propensity for peptide binding as it is well known for exposed beta-sheet edges in other proteins [18], [19], [20] and ensures productive alignment of the target hydroxyl side-chain with the bound ATP substrate.

Probably, sequence independent positioning of the backbone flanking the modifiable target residue confers an evolutionary advantage. While exposed loops in extended conformation of many proteins may easily dock to the flap, other parts of the enzyme would confer target affinity and specificity (as seen in the IbpA(FIC)/Cdc42 complex [4]) that were free to adopt during evolution without compromising on the catalytic mechanism. Notably, peptide registration to the active site via main-chain interactions is known also for serine proteases [21] and protein kinases [22], [23].

## Discussion

The vast majority of Fic proteins are characterized by a conserved HxF[D/E]GNGRxxR active site motif and catalyses adenylylation, an enzymatic activity that involves nucleophilic attack of a target hydroxyl group onto the α-phosphate of ATP. Productive AMP transfer therefore relies on the proper juxtaposition of the reaction partners. The inhibition-relieved (E->G) mutant structures of Fic proteins from the three distinct classes shed light on the importance of the active site [D/E]GNGRxxR residues to enable catalytically competent ATP substrate binding. Indeed, in the three classes, these residues, by way of a large hydrogen-bonding network, enable a unique mode of ATP binding to orientate favorably the α-phosphate relative to the target side-chain hydroxyl group (Fig. 3). The latter is registered to the FIC active site in-line with the scissile Pα-O3α bond *via* sequence-independent main chain-main chain interactions with the target dock at the edge of the FIC flap (Fig. 5). Thus, the FIC active site and the target dock are two indivisible structural elements that have been exposed to high functional constraints to ensure productive catalysis. Fic proteins with degenerated active site signature motifs and/or devoid of a flap-like structure are likely to have adopted new functions.

In Fic proteins of the three inhibition classes, the inhibitory glutamate plays the same role. It out-competes the γ-phosphate for binding to arginine R(2) of the FIC signature motif (Fig. 4). This results in an α-phosphate orientation that does not permit an attack of the incoming target side-chain hydroxyl group. Interestingly, though the active sites are structurally well conserved, the nucleotide triphosphates show variation in their binding to the Fic proteins of the three classes (Fig. 2). This is in contrast to the uniform binding mode found in the inhibition relieved mutants (Fig. 3) and shows that the mode of ATP binding to the inhibited enzyme *per se* was not under evolutionary constraints.

Knowledge of the universal catalytic and inhibitory mechanism of Fic mediated AMP transfer will now pave the way for further studies towards the physiological roles of Fic proteins and particularly the identification of their protein targets. It may also prompt rational structure based design of small molecule inhibitors targeting the ATP binding pocket or novel peptides that mimic the inhibitory helix to neutralize bacterial virulence factors which kill their host *via* uncontrolled Fic-mediated adenylylation activity.
